# Dr. John E. Register

**Published:** 1893-01

**Authors:** 


### Obituary.

### DR. JOHN E. REGISTER.

Dr. Register died in Philadelphia on November 8, 1892, of tuberculosis of the bowels, aged forty-seven.

His death, though not wholly unexpected from his failing health, came to his friends with the feeling that his work, valuable as it had been in certain directions, was not completed. He had reached an age when the enlarged experience and deeper thought promised more valuable results.

His preparatory studies in his profession were made with his brother, Dr. H. C. Register, of Philadelphia, and he graduated at the Pennsylvania College of Dental Surgery, in the class of 1869. He was always a diligent student, and succeeded in securing a special recognition from the Faculty for ability as an operator. His labors in chemical and mechanical improvements continued to the day of his death, and these he freely gave to his profession. One of the last of his productions, perhaps the last, appeared in the October number of this journal, on "Gold added to Copper Alloy." His retiring disposition made him less prominent than he should have been, but those who knew him best appreciated the modest worth of his character.

He began practice at Port Deposit, Maryland, but finally removed to Dover, Delaware.

His wife died five years ago, two daughters surviving them.

